# Synergistic Inorganic Carbon and Denitrification Genes Contributed to Nitrite Accumulation in a Hydrogen-Based Membrane Biofilm Reactor

**DOI:** 10.3390/bioengineering9050222

**Published:** 2022-05-20

**Authors:** Si Pang, Bruce E. Rittmann, Chengyang Wu, Lin Yang, Jingzhou Zhou, Siqing Xia

**Affiliations:** 1 State Key Laboratory of Pollution Control and Resource Reuse, College of Environmental Science and Engineering, Tongji University, Shanghai 200092, China; pancy@tongji.edu.cn (S.P.); chengyang@tongji.edu.cn (C.W.); jade_ylin@tongji.edu.cn (L.Y.); 1910545@tongji.edu.cn (J.Z.); 2 Shanghai Institute of Pollution Control and Ecological Security, Shanghai 200092, China; 3 Biodesign Swette Center for Environmental Biotechnology, Arizona State University, Tempe, AZ 85287, USA; rittmann@asu.edu

**Keywords:** membrane biofilm reactor, nitrite accumulation, inorganic carbon fixing genes, denitrification genes, microbial community, autotrophic partial denitrification

## Abstract

Partial denitrification, the termination of NO_3_^−^-N reduction at nitrite (NO_2_^−^-N), has received growing interest for treating wastewaters with high ammonium concentrations, because it can be coupled to anammox for total-nitrogen removal. NO_2_^−^ accumulation in the hydrogen (H_2_)-based membrane biofilm reactor (MBfR) has rarely been studied, and the mechanisms behind its accumulation have not been defined. This study aimed at achieving the partial denitrification with H_2_-based autotrophic reducing bacteria in a MBfR. Results showed that by increasing the NO_3_^−^ loading, increasing the pH, and decreasing the inorganic-carbon concentration, a nitrite transformation rate higher than 68% was achieved. Community analysis indicated that *Thauera* and *Azoarcus* became the dominant genera when partial denitrification was occurring. Functional genes abundances proved that partial denitrification to accumulate NO_2_^−^ was correlated to increases of gene for the form I RuBisCo enzyme (*cbbL*). This study confirmed the feasibility of autotrophic partial denitrification formed in the MBfR, and revealed the inorganic carbon mechanism in MBfR denitrification.

## 1. Introduction

Denitrification is an important process in biological treatment of water and wastewater [1]. In most cases, complete denitrification of nitrate (NO_3_^−^) to dinitrogen gas (N_2_) is the desired outcome, as it brings about total-nitrogen (TN) removal. Partial denitrification occurs when the reduction stops at intermediate products (nitrite or nitrous oxide). Although partial denitrification is an undesired outcome in most situations [2], it may be the desired result in special cases, such as when nitrite (NO_2_^−^) is the electron accepter for anammox [1,3,4] or nitrous oxide (N_2_O) is recovered as a highly energetic oxidant, such as for rocket fuel [5].

One cause of partial denitrification is a pH greater than about 9 [6]. Because denitrification produces approximately one equivalent of strong base for one equivalent of N reduced when the extent of reduction goes beyond nitrite, complete denitrification can lead to a pH increase if the acid is not great enough to buffer the pH [7].

Controlling the extent of denitrification has been investigated to a limited extent for heterotrophic systems [4], but not for the Hydrogen-Based Membrane Biofilm Reactor (H_2_-MBfR). One advantage of the H_2_-MBfR in this context is that the delivery capacity of the electron donor (H_2_ gas) is regulated by the H_2_ regulator to the hollow fiber membranes [8,9]. The maximum delivery flux can be determined by the membrane type and the H_2_ pressure [8].

**Figure 1:**
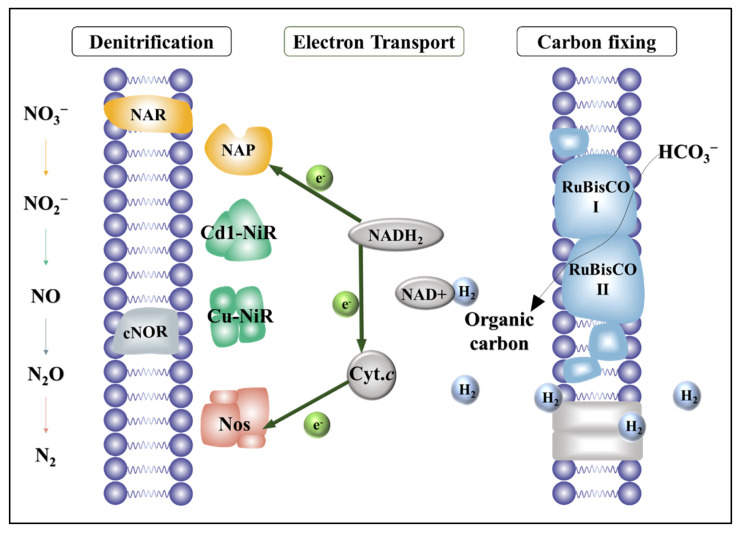
Schematic of the respiratory chain of denitrification. Electrons are transferred from NADH_2_ to NO_x_ reductases (i.e., nitrate, nitrite, nitric oxide and nitrous oxide reductases).

An important factor is that NO_3_^−^ reduction is a stepwise process carried out by different reductase enzymes. Figure 1 summarizes the steps and their reductases. First, NO_3_^−^ receives electrons from the intracellular electron donor (NADH_2_) and is reduced to NO_2_^−^ by either a membrane-bound nitrate reductase (NAR) or a periplasmic nitrate reductase (NAP) [10], which are encoded by genes *narG* and *napA*, respectively [11]. NO_2_^−^ is further reduced to NO through nitrite reductase (NIR), and *nirK* and *nirS* are the most widely used genes [12]. Next, *cnorB* genes encode cNOR proteins that catalyze NO reduction to N_2_O [13]. Finally, N_2_O is reduced to N_2_ by nitrous oxide reductase (*NosZ*) [14], which is encoded by gene *nosZ* [15].

Another important method for achieving partial denitrification is choosing a suitable carbon source (such as glucose [16], ethanol [17], or methanol [18]) and controlling the carbon ratio (COD/ NO_3_^−^-N) [19]. For the H_2_-MBfR, autotrophic bacteria have an important electron demand to reduce inorganic carbon (IC, i.e., H_2_CO_3_ and HCO_3_^−^) as the carbon source [20]. Electron demand by the NO_x_ reductases competes with the electron demand for reducing inorganic carbon for synthesis. Although five carbon-fixation pathways are known [21], the Calvin cycle is the main pathway for autotrophic denitrifiers [20]. RuBisCO, the key enzyme in Calvin cycle for fixing CO_2_ [20], has two main forms—RuBisCOI and RuBisCOII—in autotrophic bacteria. Although both catalyze the first step of the Calvin cycle, RuBisCOI and RuBisCOII possess different catalytic features, such as lower CO_2_/O_2_ substrate specificity and poorer affinity for CO_2_ for RuBisCOII [22]. RuBisCOI is encoded by gene *cbbL*, and RuBisCOII is encode by gene *cbbM* [23]. So far, the *cbbL* and *cbbM* genes have been used to indicate the diversity of environmental community [24]. Though soil environmental factors (including organic matter, pH, nitrogen, and salinity) influenced their abundances [25], their variations of abundance in MBfRs are not clear.

Although some studies with H_2_-MBfRs have reported the accumulation of nitrite for different nitrate surface-loading rates and H_2_ pressures [9,26], the mechanisms for controlling the extent of denitrification have not been explored. Therefore, we systematically investigated the first step of partial denitrification based on molecular-level analyses. We applied quantitative real-time PCR (qPCR) to analyze changes in each stage according to six denitrifying genes—*narG*, *napA*, *nirK*, *nirS*, *cnorB*, and *nosZ*—and inorganic carbon fixing functional genes—*cbbL* and *cbbM*. We also tracked community changes occurred during different stages using 16S rRNA high-throughput amplicon sequencing.

## 2. Materials and Methods

### 2.1. Experimental Setup

Figure S1 provides a schematic of the H_2_-MBfR used in this study. The total working volume was 60 mL, and the main column contained 30 hollow-fiber membranes (non-porous polypropylene fiber, 200 μm OD, 100 μm ID, wall thickness 50 μm; Teijin, Ltd., Tokyo, Japan) with a length of 28 cm. High-purity H_2_ was supplied to these hollow fiber membranes through a gas pipeline connected to a H_2_-gas tank, and the pressure was regulated by a regulator at the end of the gas pipeline.

The influent flow rate was regulated at 0.27 mL min^−1^ in this study by a peristaltic pump (BT100-2J, Longerpump, Baoding, China). The reactor was well mixed using a peristaltic pump (BT100-2J, Longerpump, China) with the recirculation rate of 81 mL/min so that the effluent concentration was equal to solute concentration inside the reactor. In order to keep the influent O_2_-free, a gas bag filled with N_2_ was connected to the influent tank.

### 2.2. Biomass Enrichment

Biomass was collected from an anoxic tank in the Quyang wastewater treatment plant (Shanghai, China) and cultured with NO_3_^−^ and H_2_ for over a week to enrich autotrophic denitrifiers before inoculating the MBfR. The medium for enrichment culturing contained NaNO_3_ (10 mg/L N), NaHCO_3_ (60 mg/L), phosphate buffer (0.4 mM, pH = 7.4), and trace elements [27]. H_2_ was injected to culture bottle through gas injector as the sole electron donor, HCO_3_^−^ was the sole inorganic carbon source for the growth of autotrophic microorganisms, and NO_3_^−^ was the sole electron acceptor and nitrogen source [26]. N_2_ was sparged to the medium to remove dissolved oxygen before H_2_ was delivered by a gas bag connected through a plastic pipe. Once the concentration of NO_3_^−^ decreased below the detection limit (~0.32 mg N/L), more medium was added, eventually achieving the enriched hydrogen autotrophic denitrifying inoculants for the H_2_-MBfR.

### 2.3. Synthetic Medium and MBfR Operation

The influent contained NaNO_3_ (variable), NaHCO_3_ (variable), phosphate buffer (0.4 mM, pH = 7.4), and trace elements [27]. NaNO_3_ and NaHCO_3_ were added according to Table 1 to achieve the desired concentrations for each stage.

The operating factors of each stage are summarized in Table 1. At the start of Stage 0-1, the H_2_-MBfR was inoculated with 20 mL of suspended and enriched hydrogen-autotrophic denitrifying bacteria from the enrichment culture described in Section 2.2; then, the influent was stopped for two days to form a biofilm on the surface of hollow fiber membranes. Subsequently, the MBfR was fed continuously with influent for 15 days until no NO_3_^−^-N was detected in the effluent.

For Stages 0-1 (day 0–13), 0-2 (day 14–25), and 0-3 (day 26–50) and for Stage 1 (51–80), the influent pH was 7.4, and the effluent pH increase was due to base production during denitrification [6]. For Stage 2 (day 81–95), Stage 3 (day 96–121) and Stage 4 (day 122–146), the pH was adjusted with 1-M NaOH as needed in order to keep the MBfR’s pH stable within an operation stage despite changes to the extent of denitrification.

### 2.4. Sampling and Analyses

Samples from the influent and effluent were collected on a daily basis and immediately filtered through a 0.22-μm disposable Millipore filter with a polyether sulfone (PES) membrane; the pH was measured immediately after filtration. All the liquid samples were kept in the refrigerator at 4 °C until analysis, which was within three days. The concentrations of NO_3_^−^-N and NO_2_^−^-N were analyzed by *Standard Methods* [28]. The pH and DO were measured by a combined pH and DO meter (HQ40d, HACH, Loveland, CO, USA). Inorganic carbon measurements were performed with a total organic carbon (TOC) analyzer (TOC-L, Shimadzu, Kyoto, Japan).

Biofilm samples were collected at the ends of Stages 0–1, 1, 2, 3, and 4. For each sampling, the fiber bundle was taken from the tube, and an ~12-cm-long section of a coupon fiber was cut using a sterilized scissors. Then, a knot was tied at the end of the remaining fiber before the fiber bundle was return to the reactor. The biofilm-containing fiber samples were cut into pieces and immediately stored in a sterilized centrifuge tube at −80 °C.

### 2.5. Alkalinity Calculation and Electron-Equivalent Fluxes Analysis

The effluent alkalinity and the maximum delivery fluxes of H_2_ was computed with the model of Tang et al. [6] and alkalinity inputs to the model were pH, the influent concentration of NO_3_^−^, and the effluent concentrations of NO_3_^−^ and NO_2_^−^, all of which were measured.

The NO_3_^−^-N surface loading (SL) and maximum NO_3_^−^-reduction consumption flux (J_NO3_^−^) were determined by the methods of Xia et al. [29]:

(1) $$\mathrm{SL}=\frac{{\mathrm{QS}}^{0}}{\mathrm{aV}}$$

(2) $$J_{{{\mathrm{NO}}_{3}}^{-}}=\frac{Q\alpha \left(S^{0}-S\right)}{\mathrm{aV}}$$

where Q is the influent flow rate in m^3^/d and S^0^ is the influent concentration of the acceptor (g N/m^3^). a is the specific surface area (m^−1^), and V is the reactor volume (m^3^). The value of α depends on the extent to which NO_3_^−^ is reduced: the ratios (in g H_2_/gN) are 0.36, 0.29, 0.21, and 0.14 for reductions to N_2_, N_2_O, NO, and NO_2_^−^, respectively, when biomass synthesis is not considered. Biomass synthesis increases the ratios: e.g., the value of α with biomass synthesis is around 0.41 g H_2_/gN for reduction of NO_3_^−^ to N_2_ [30].

### 2.6. Inorganic Carbon for Synthesis in Full Denitrification and Partial Denitrification

The synthesis removal of IC was estimated based on full versus partial nitrate reduction. The stoichiometry of IC consumption is shown in Equation (3) for full denitrification to N_2_ and in Equation (4) for partial denitrification to NO_2_^−^, based on net synthesis (original synthesis plus endogenous decay) [30]:

Full denitrification to N_2_:

0.5H_2_ + 0.1773NO_3_^−^ + 0.0246CO_2_ + 0.1773H^+^ = 0.00493C_5_H_7_O_2_N + 0.0862N_2_ + 0.5714H_2_O (3)

Partial denitrification to NO_2_^−^:

0.5H_2_ + 0.4359NO_3_^−^ + 0.0246CO_2_ + 0.00493H^+^ = 0.00493C_5_H_7_O_2_N + 0.431NO_2_^−^ + 0.4852H_2_O (4)

in which hydrogen gas (H_2_) is the autotrophic electron donor, CO_2_ is the inorganic carbon (IC) source, and biomass is indicated by C_5_H_7_O_2_N. Because Equations (3) and (4) are written for one electron equivalent of H_2_ (i.e., 0.5 mol H_2_), the amount of NO_3_^−^ consumed is much less for full denitrification (0.1773 mol, Equation (3)) than that for partial denitrification to NO_2_^−^ (0.4359 mol, Equation (3)). Thus, the ratio of IC consumed to NO_3_^−^ consumed is much greater for full denitrification: 0.12 vs. 0.05 gC/gN for full and partial denitrification, respectively.

### 2.7. DNA Extraction and qPCR Analysis

The biofilm attached on a fiber was extracted directly from the fiber using a PowerBiofilm DNA Isolation kit (Mo Bio Laboratories, Carlsbad, CA, USA) according to the manufacturer’s instructions; the DNA concentration was measured by NanoDrop 2000 (Thermo Scientific, Waltham, MA, USA). The quantitative polymerase chain reaction (qPCR) using a CFX96 Touch real-time PCR Detection System (Bio-Rad, Herculers, CA, USA) with the fluorescent dye SYBR-Green approach was employed for amplifying the functional genes. The denitrification functional genes were periplasmic nitrate reductase (*napA*), membrane-bound nitrate reductase (*narG*), cytochrome cd1 nitrite reductase (*nirS)*, copper nitrite reductase (*nirK*), nitric oxide reductase (*cnorB*), and nitrous oxide reductase (*nosZ*) genes. The functional genes for fixing inorganic carbon were for ribulose-1,5-bisphosphate carboxylase/oxygenase (RuBisCO): *cbbL* and *cbbM*. The primer sequences are provided in Table S1.

The qPCR amplification was performed in 20-μL reaction mixtures containing 10 μL of iTagTM Universal SYBR Green Supermix (Bio-Rad), 1 μL of template DNA (sample DNA or plasmid DNA for standard curves), 1 μL of forward and reverse primers respectively, and 7 μL of ddH_2_O. The qPCR protocol involved 35–40 cycles. The results of qPCR were normalized by DNA concentration for the comparing of different DNA samples.

### 2.8. 16S rRNA Sequencing and Data Analysis

DNA samples were sent to Shanghai Majorbio Technology (Shanghai, China) for Illumina MiSeq sequencing with standard protocols. Primers 338F (5′-ACTCCTACGGGAGGCAGCA-3′) and 806R (5′-GGACTACHVGGGTWTCTAAT-3′) were used to amplify the conserved V3-V4 regions for sequencing the 16S rRNA gene. Other steps were followed the procedures of sequencing described by Xia et al. [31].

Community structure and the similarity of biofilm samples were analyzed with Principal Co-ordinates Analysis (PCoA). A redundancy analysis (RDA) was performed using CANOCO 5 (Version 5.02, Petr Šmilauer, České Budějovice, Czech Republic) to explore the correlation between environmental factors and key functional gene distribution.

## 3. Results and Discussions

### 3.1. Reactor Performance

**Figure 2:**
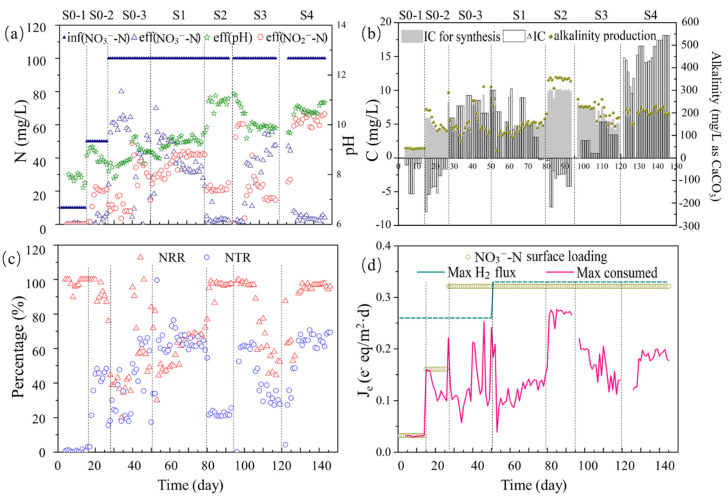
Performance of the MBfR for treatment stages S0-1, S0-2, S0-3, S1, S2, S3, and S4: (**a**) Concentrations of influent NO_3_^−^-N and effluent NO_3_^−^-N and NO_2_^−^-N, along with pH; (**b**) Concentration of IC used for synthesis and changes in IC and alkalinity. ΔIC is defined as (Inf.IC − Eff.IC); (**c**) NRR (nitrate removal ratio), defined as (Inf.NO_3_^−^ − Eff.NO_3_^−^)/Inf.NO_3_^−^; and NTR (nitrate transformation ratio), defined as Eff.NO_2_^−^/(Inf.NO_3_^−^ − Eff.NO_3_^−^); (**d**) calculated maximum H_2_ flux, NO_3_^−^-N surface loading, and maximum NO_3_^−^-reduction consumption flux (all in e^−^ eq/m^2^·d).

Electron demand by the NO_x_ reductases competes with the electron demand for reducing inorganic carbon for synthesis. Hence, the form and concentration of both nitrogen and inorganic carbon were considered in this study. Figure 2 summarizes MBfR performance in terms of nitrate concentration, inorganic carbon concentration, alkalinity concentration, pH, and electron-donor and -acceptor fluxes for the experimental duration of 146 days. In solution, carbonic acid (H_2_CO_3_) dissociates (p*K*_a1_ = 6.1, 25 °C) to yield a proton and bicarbonate (HCO_3_^−^), and bicarbonate dissociates to carbonate (CO_3_^2−^) (p*K*_a2_ = 9.9, 25 °C) [32]. Although the inorganic carbon added in this H_2_-MBfR was HCO_3_^−^, the evaluated concentration of H_2_CO_3_, HCO_3_^−^ and CO_3_^2−^ at each stage were shown in Figure S2. For analyzing IC variation, the Figure S3 also provides the average removal of inorganic carbon in each stage. In general, no organic carbon sources added during reactor operation, which showed obvious economization compared with some heterotrophic reactor [4,33,34].

The first 50 days (Stage 0) were for biofilm accumulation, and the maximum H_2_ flux was maintained at 0.26 e^−^ eq/m^2^·d (Figure 2d). Then, the influent NO_3_^−^-N concentration was increased stepwise from 10 (S0-1) to 50 (S0-2) and again to 100 (S0-3) mg N/L; at the same time, the influent IC was increased in the same proportion (Figure 2a and Figure S3). Throughout Stage 0, the NO_3_^−^-transformation ratio (NTR, accumulated NO_2_^−^-N/consumed NO_3_^−^-N) increased from 0 to 60%, but the NO_3_^−^-removal ratio (NRR, consumed NO_3_^−^-N /influent NO_3_^−^-N) decreased from 100% in S0-1 to 47% by the end of S0-3 (Figure 2c). These trends can be attributed to the actual H_2_ flux approaching the maximum H_2_-delivery capacity in stage S0-3 (Figure 2d).

In order to alleviate the limitation of H_2_ on denitrification, the H_2_ gauge pressure was increased from 0.04 to 0.08 MPa in Stage S1, and the NRR increased to 77% by the end of S1 (Figure 2c). However, the effluent IC did not change significantly (Figure S2), and synthesis and alkalinity production also did not significantly change (Figure 2b). This means that the increase in electron donor supply did not alter the carbon metabolic of the bacteria, but led to more removal of nitrate.

In order to investigate the influence of pH on partial denitrification, the effluent pH was adjusted from 9.25 ± 0.28 to 10.85 ± 0.33 in S2. During this stage, the removal efficiency of influent NO_3_^−^-N was >90%, while the NTR was in the range of 21 to 26% (Figure 2c), which indicates that most of the NO_3_^−^-N consumed was converted to N_2_. The maximum NO_3_^−^-consumption flux increased to about 0.27 e^−^ eq/m^2^·d, a value close to the NO_3_^−^-N loading flux (0.32 e^−^ eq/m^2^·d). However, the effluent IC became higher than the influent IC, while synthesis and alkalinity reached maxima (Figure 2b). The increase in effluent IC and alkalinity was from biomass decay, probably due to the higher pH. Furthermore, higher pH led to the dissociation of bicarbonate, which increased the concentration of CO_3_^2−^ up to the range of 349–407 mg/L, while the concentration of HCO_3_^−^ declined to 60–92 mg/L (Figure S2b). At this stage, the alkalinity production reached the maximum, which could result in membrane fouling in MBfR [35], further influence the nitrogen removal rate [36]. However, the NRR in this stage still higher than 90% when partial denitrification occured. Hence, another advantage of this technology was to reduce the influence of membrane fouling on reactor performance.

The IC concentration in the influent was decreased to 43 mg/L to explore the effect of inorganic carbon on denitrification in S3. After that, the pH was adjusted to be close to the pH in S1 (Figure 2a), which caused the concentration of HCO_3_^−^ to become higher than CO_3_^2−^ again (Figure S2b). The NTR and NRR decreased by the end of S3, as did synthesis and alkalinity production (Figure 2b,c). However, at the beginning of this stage, when the pH had not yet been decreased, the NRR was nearly 100% and the NTR increased to 60%. Thus, decreasing the pH led to a degradation in performance.

To determine if a high NO_2_^−^-N accumulation rate could be restored following an increase in pH, a fourth stage (S4) was undertaken in which the pH in the effluent was increased to 10.4–10.9 (Figure 2a). The NTR increased to about 68% (Figure 2c), which was similar to what was observed at the beginning of S3, and the effluent IC dropped to about 30 mg/L in parallel with the increase of NTR (Figure 2c). At the same time, NRR stabilized at about 95%, achieving a good performance compared with other heterotrophic partial denitrification reactors [33,37]. In addition, the maximum consumed electron equivalents from H_2_ decreased to 0.11–0.19 e^−^ eq/m^2^·d, far lower than the maximum H_2_ flux (Figure 2d). Thus, H_2_ consumption declined significantly due to partial denitrification. Furthermore, the evaluated concentration of HCO_3_^−^ reached a minimum, although synthesis and alkalinity production did not change significantly. As a whole, a higher pH than common partial denitrification reactors [34,38] provied more posibilities for the applications of this technology. Besides, lower electron consumption campared to previous researches [29,36] not only improved the nitrate removal capacity but also reduced H_2_ safety risks, providing a possibility for the widespread application of H_2_-MBfR.

### 3.2. N- and Electron-Flow Balances for Full Denitrification vs. Partial Denitrification

**Figure 3:**
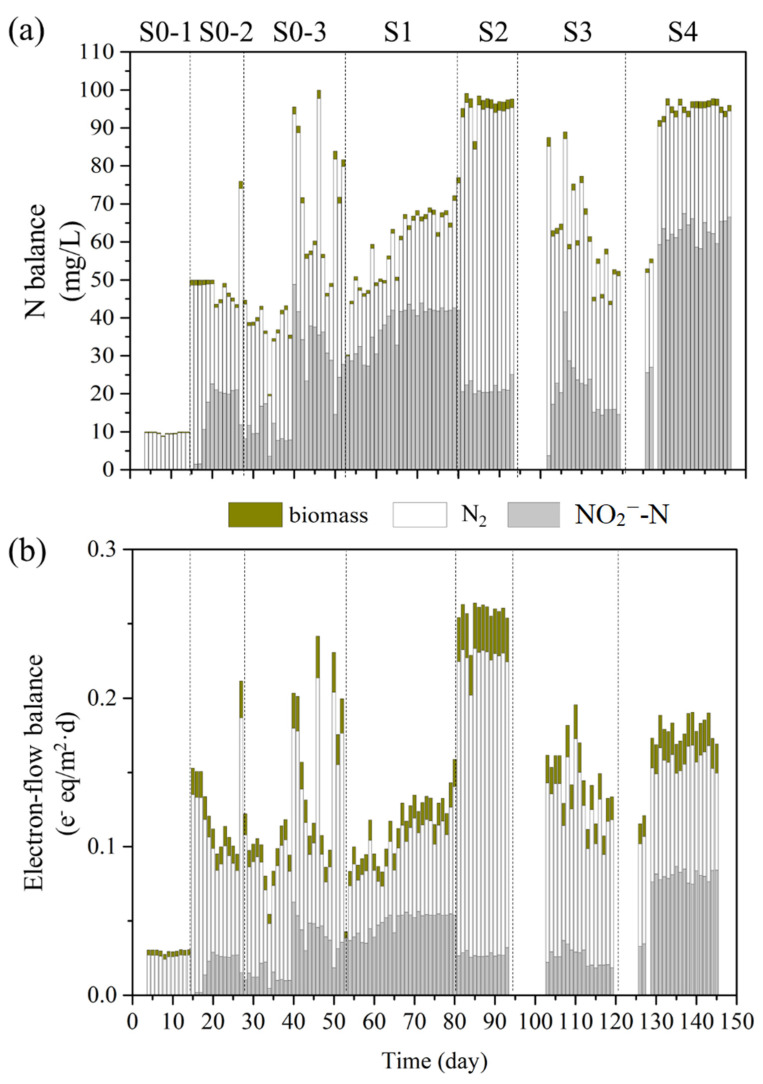
The N balance (**a**) and electron-flow balance (**b**), along with biomass synthesis (**a**,**b**) in full and partial denitrification.

Since the fate of nitrogen determines whether the denitrification is full or partial, N-flow (Figure 3a) and electron-flow (Figure 3b) balances were calculated to show the distributions of N from NO_3_^−^ and electrons from H_2_ into NO_2_^−^-N, N_2_, and biomass. Their percentages are shown in Figure S3. In general, biomass synthesis always was a small percentage of electron consumption, less than 12% (Figure S3b).

During the biofilm-accumulation stage (first 50 days), with the stepwise increase of influent NO_3_^−^-N, the N flow of NO_3_^−^-N going to N_2_ increased from 9 to 62 mg/L. That’s the result of enough biomass for full denitrification. But limited by H_2_, the percentage of NO_3_^−^-N to N_2_ decreased from 97% to 32%, and its electron flow decreased from 88% to 43% (Figure S3). At the same time, the percentage of NO_3_^−^-N going to NO_2_^−^-N increased from 0% to 66%, and its electron flow increased from 0% to 46%.

After increasing the H_2_ supply, both of the N- and electron- flows to NO_2_^−^-N and N_2_ were all increased (Figure 3) modestly in stage S1. However, the percentages of N balance and electron-flow balance showed that the N- and electron- flows to N_2_ decreased, and the flows to NO_2_^−^-N increased (Figure S3). What’s more, the nitrate transformation ratio (NTR) increased from 34% to 67% (Figure 2c). Sufficient H_2_ supply provided adequate electron donors for denitrification, thus the partial denitrification can be improved. Although the shortage of electron donors is one way to achieve partial denitrification, securing an adequate supply of electron donors is equally important. Hence, allowing the actual H_2_ flux to approach the maximum delivery flux was the best choice and the next stages proved this.

On account of the high pH in S2, the N balance of NO_3_^−^-N going to N_2_ increased significantly, with the flow of NO_3_^−^-N going to NO_2_^−^-N decreased; the trends were the same for electron flow. High pH changed the form of inorganic carbon from HCO_3_^−^ to CO_3_^2−^ (Figure S2b) and the synthesis reached maxima (Figure 2b), which caused the denitrification more reduced than NO_2_^−^-N.

In S4, the decreasing influent IC and increasing pH led to stepwise decreases in flows to N_2_, while flows to NO_2_^−^-N increased up to 68 mg/L. Compared with S2, lower concentration of influent IC resulted lower concentration of HCO_3_^−^ and electrolytic CO_3_^2−^, which caused lower N converted to biomass synthesis in S4 (1.6–1.8%).

N- and electron-flow balances showed that the electronic competition and carbon metabolism were influenced. Further studies on inorganic carbon fixing genes and denitrifying genes explained the mechanism on partial denitrification.

### 3.3. Synergy of Functional Genes

Although the N- and electron-flow balances quantify full versus partial denitrification, they do not indicate the mechanisms for the changes. Mechanistic insights can be gained from the changes in relative abundances of six denitrifying genes and two inorganic carbon fixing genes, which were measured using qPCR.

**Figure 4:**
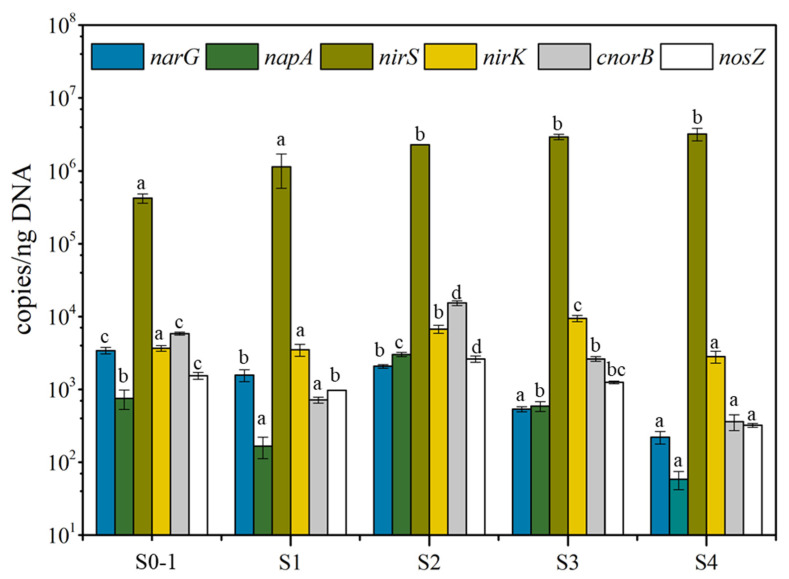
Relative abundances of six denitrification genes in biofilms in each stage. Different lower-case letters indicate significant differences among stages at *p* < 0.05.

The relative abundances of six denitrifying genes, reported in Figure 4, show that *nirS* (a cytochrome-based nitrite reductase) was by far the most prevalent gene, and its abundance gradually increased, from 4.2 × 10^5^ copies/ngDNA to 3.2 × 10^6^ copies/ngDNA, over the stages. The *nirS* gene codes for an enzyme that reduces NO_2_^−^ to NO, the second step of denitrification [39]. All other genes were at least two orders of magnitude fewer and appeared to become less prevalent in S3 and S4 (5.9 × 10^1^–1.5 × 10^4^ copies/ngDNA). These trends suggest that nitrite should be reduced readily in normal circumstances because of the high abundance of the *nirS* nitrite reductase, which is not inhibited by high pH [40]. Genes encoded nitrate reductases (*narG* and *napA*) showed lower abundances compared with *nirS*. However, the abundance of *narG* in heterotrophic reactor [4,34] always higher than other denitrification genes. Hence, the nitrite accumulation achieved easier in heterotrophic reactor due to the restriction on the electron flow to *NirS* [34]. Even so, autotrophic partial denitrification achieved in this study under high pH.

Long-term exposure of bacteria to high pH significantly inhibits the activity of copper-type nitrite reductase (*NirK*) [40]. Consistent with this, the N- and electron-flow balances (Figure 3) show that the relatively lower abundance of *nirK* in S1 (3.5 × 10^3^ copies/ngDNA) and S4 (2.8 × 10^3^ copies/ngDNA) correlated with the highest flows of N and electrons going to NO_2_^−^-N. Metagenomics [4] also find *nirS* increased and *nirK* decreased when partial denitrification occurred.

Nitrous oxide reductase (*NosZ*) catalyzes the terminal step in canonical denitrification, the reduction of NO to N_2_ [14]. The abundance of *nosZ* was low in S1 (9.7 × 10^2^ copies/ngDNA) and S4 (3.2 × 10^2^ copies/ngDNA), compared with S2 (2.6 × 10^3^ copies/ngDNA) and S3 (1.3 × 10^3^ copies/ngDNA), which has the least flows of N and electrons to N_2_. Thus, the abundances of denitrifying genes may have played roles in determining the balance of full versus partial denitrification.

**Figure 5:**
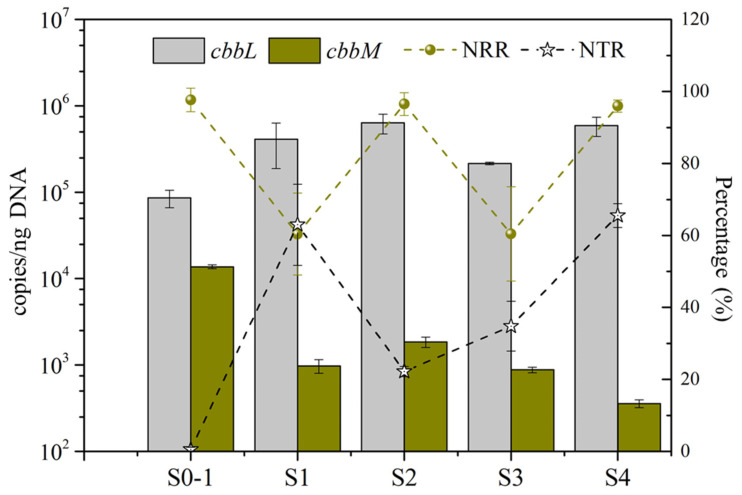
Abundances of gene *cbbL* and *cbbM*, as well as the nitrate-removal ratio (NRR) and nitrite-transformation ratio (NTR), during each stage.

The Calvin cycle has the highest C-fixation efficiency for autotrophs to assimilate CO_2_ and is widely present in bacteria [22]. A previous study showed a link between the Calvin cycle and nitrogen fixation, which is another electron-demanding process [41]. Figure 5 summarizes the abundances of the *cbbL* and *cbbM* genes over the stages. In general, the abundance of *cbbL* (8.6 × 10^4^–6.4 × 10^5^ copies/ngDNA) was at least one order of magnitude greater than for *cbbM* (3.4 × 10^2^–1.4 × 10^4^ copies/ngDNA), which highlights the importance of *cbbL* in carbon fixation in the MBfR biofilm. Furthermore, the abundance of *cbbM* mostly went in the opposite direction to that of *cbbL*, which means they were not expressed together, as a previous study showed [42]. Its greater affinity for HCO_3_^−^ [22] may explain why *cbbL* was dominant.

The abundance of *cbbL* mirrored the trends for the electrons flow to biomass synthesis in Figure 3b, which showed a competition for electrons when the donor is in short supply. Because the cells may produce more *cbbL* to get scarce electrons to biomass synthesis, the abundance of *cbbL* also mirrored the trends for nitrate-removal rate (NRR) in Figure 5, except for S0-1, when the influent nitrate concentration was only 10 mg N/L, compared to 100 mg N/L for S1–S4. Thus, when the NRR was larger, the cells may have compensated by producting more *cbbL*.

Furthermore, the abundance of gene *cbbM* had an inverse correlation with nitrite transformation ratio (NTR). Higher NRR and NTR in S4 means that partial denitrification was achieved by increasing pH and decreasing the IC concentration, both of which make the H_2_CO_3_ concentration low. This was associated with high abundance *cbbL* and low abundance of *cbbM*.

### 3.4. Microbial Community Characterizations of the Biofilms

In this study, a community with a high rate of NO_2_^−^ accumulation via autotrophic partial denitrification was enriched in an electron donor (H_2_)-limited continuous membrane biofilm reactor (MBfR) through varying the NO_3_^−^-N concentration, inorganic-carbon concentration, H_2_ pressure, and pH. By systematically analyzing the differences of community structure in each stage, the methods and mechanism for enriching partial denitrifying bacteria was discussed.

**Figure 6:**
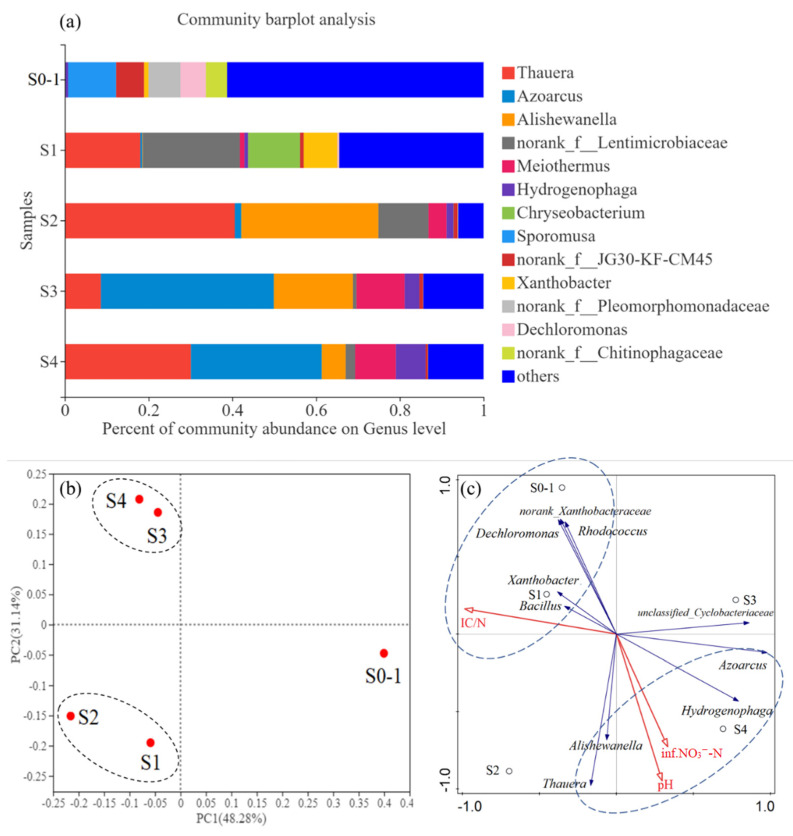
Distribution of the bacterial community at genus level (>5%) (**a**) and principal coordinates analysis of them during different stages of reactor operation (**b**), as well as the Redundancy analysis (RDA) map of the response of known denitrifiers to environmental factors (**c**).

The microbial community structures (Figure 6a) and PCoA analysis based on the OTUs (Figure 6b) show that the community in S0-1 was distinct from other stages, and the communities in S1 and S2 were similar to each other, as were the communities of S3 and S4. The overlap of communities in S1 and S2 (and also S3 and S4) is expected, since both correlated stages had the same influent concentration of IC: For S1 and S2, the IC was 86 mg C/L, versus 43 mg C/L for S3 and S4 (Table 1).

Known denitrifers—genera *Azoarcus*, *Thauera*, *Hydrogenophaga*, *Alishewanella*, *Bacillus*, unclassified_*Cyclobacteriaceae*, norank_*Xanthobacteraceae*, *Xanthobacter*, *Dechloromonas*, and *Rhodococcus*—accounted for up to 41.3% of total reads (Table S3). A dominant genus was *Thauera*, a member of the γ-*Proteobacteria* and a functional bacterium for partial denitrification [3], increased from 0% of the total bacteria at the start of stage 0 (stage 0–1), to 18.0% during the H_2_ pressure increase in stage 1. During stage 2 the relative abundance of *Thauera* increased to 40.6%, significantly higher than stage 1 due to the higher of pH. However, a falling down occurred in pH in the end of stage 3 and the relative abundance of *Thauera* decreased to 8.6%. When the pH levels were restored in stage 4 the *Thauera* increased to 30.1%. In other partial deinitrificaiton reactors, *Thauera* was the dominant functional bacteria too [3,16], and was identified to be responsible for nitrate reduction [34]. Recently, the regulatory network of denitrification has been studied in detail for a few model organisms, revealing a large number of transcriptional regulator enzymes and ancillary factors as well as a variety of denitrification regulatory phenotypes [39]. In most cases it has been observed that NO_2_^−^ only accumulates transiently during denitrification. A notable exception is several species of *Thauera* which exhibited a progressive onset of denitrification where NO_2_^−^ accumulated quantitatively from available NO_2_^−^ before further reduction to gaseous end products [43]. However, not all *Thauera* show a progressive onset denitrification phenotype since other strains reduce NO_3_^−^ directly to gaseous intermediates without NO_2_^−^ accumulation [43]. Thus, a further exploration on functional genes conducted in this study and proved that inorganic carbon fixing genes (*cbbL* and *cbbM*) played an important role in a progressive onset of denitrification.

Although culturing for specific functional bacteria is necessary, the microbial community structures (Figure 6a) proved that a high-performance partial denitrification was the result of joint action of various denitrifiers. The genus *Azoarcus* occurred in 31.2–41.3% was more abundant in the stages with influent IC/N 0.4 (S3 and S4) than those with influent IC/N 0.9 (S0, S1 and S2). Similar with this study, *Azoarcus* also dominated in a partial denitrification driven by salinity [4], even though it was a heterotrophic reactor. This was attributed to similar initial community structure, and proved that *Azoarcus* adapted to environmental changes. Notably, the relative abundance of *Hydrogenophaga* become higher along with the operation days of this H_2_-bases membrane biofilm reactor; to the contrary, the abundance of *Alishewanella* decreased. Both *Alishewanella* and *Hydrogenophaga* were infrequent in previous partial denitrification reactors [4,38], which showed H_2_ autotrophic ability of them when partial denitrification occured.

The RDA results in Figure 6c show that *Bacillus*, *Xanthobacter*, norank_*Xanthobacteraceae*, *Dechloromonas*, and *Rhodococcus* were positively correlated with the influent IC/N ratio, whereas *Thauera*, *Alishewanella*, *Azoarcus*, and *Hydrogenophaga* were affected by pH and influent nitrate concentration. Although *Bacillus*, *Xanthobacter*, norank_*Xanthobacteraceae*, *Dechloromonas*, and *Rhodococcus* were important denitrifying bacteria, they were abundant only during full denitrification (S0-1 and S1). In contrast, *Thauera*, *Alishewanella*, *Azoarcus*, and *Hydrogenophaga* were more abundant in partial denitrification (S4). Pearson analysis was further conducted to presesnt the correlation of these four functional genera with functional genes (Table 2). *Thauera*, *Azoarcus*, and *Hydrogenophaga* were positively related with NTR. Further, NTR was negatively related with *cbbM* and *nirK*, which showed the same result with the analysis above.

Both community and gene level assessment indicated that high pH and low inorganic carbon were observed to shape the bacterial community, enriching for NO_2_^−^ accumulators. Specifically, the genus *Thauera* dominated in the high pH conditions and showed statistically significant positive correlation with *cbbL* (Table 2). Thus, conditions leading to partial denitrification selected for certain denitrifiers, which corresponded to enrichment of CO_2_-fixing *cbbL*. Recently, a study focused on carbon metabolism revealed ACS enzymes for acetate fixing contributed to heterotrophic partial denitrification [34], which was the same to *cbbL* attributed to autotrophic partial denitrification in this study.

## 4. Conclusions

Increasing pH and decreasing inorganic carbon concentration led to more partial denitrification. Biofilm-community structure and key functional genes revealed that high pH increased the abundance of *Thauera*, resulting in higher abundance of Calvin Cycle gene *cbbL* contributed to partial denitrification. The technology of partial denitrificaiton in H_2_-MBfR not only saves cost but also provids a possibility for the widespread application of H_2_-MBfR.

## Figures and Tables

**Table 1:** Operational parameters for all stages and sub-stages.

Stage	Influent NaNO_3_-N (mg/L)	Influent NaHCO_3_-C (mg/L)	H_2_ Gauge Pressure (MPa)	Effluent pH
S0-1	10	9	0.04	7.86 ± 0.18
S0-2	50	43	0.04	8.75 ± 0.25
S0-3	100	86	0.04	8.68 ± 0.44
S1	100	86	0.08	9.25 ± 0.28
S2	100	86	0.08	10.85 ± 0.33
S3	100	43	0.08	9.93 ± 0.09
S4	100	43	0.08	10.62 ± 0.17

Notes: Absolute pressure = gauge pressure + 0.101 MPa, and 1 MPa = 9.87 atm.

**Table 2:** Pearson correlation between NRR, NTR, functional bacteria and functional genes.

	*Thauera*	*Azoarcus*	*Alishewanella*	*Hydrogenophaga*	NRR	NTR	*cbbL*	*cbbM*	*nirK*
*Thauera*	1	−0.065	0.594	0.325	0.322	0.383	0.982 **	−0.633	−0.040
*Azoarcus*		1	0.136	0.772	−0.289	0.354	−0.020	−0.473	0.451
*Alishewanella*			1	0.009	0.119	−0.239	0.453	−0.387	0.723
*Hydrogenophaga*				1	0.182	0.552	0.411	−0.483	−0.089
NRR					1	−0.410	0.264	0.447	−0.407
NTR						1	0.537	−0.790	−0.286
*cbbL*							1	−0.691	−0.155
*cbbM*								1	−0.275
*nirK*									1

** significant at 1% level.

## Data Availability

Not applicable.

## Supplementary Materials

The following supporting information can be downloaded at: https://www.mdpi.com/article/10.3390/bioengineering9050222/s1, Figure S1: Schematic of the H_2_-based MBfR using polypropylene hollow-fiber membranes; Figure S2: (a) Concentration of influent IC and alkalinity, along with effluent IC and alkalinity at each stage. (b) Evaluated concentration of H_2_CO_3_, HCO_3_^−^ and CO_3_^2−^ at each stage; Figure S3: The percentages of N balance (a) and electron-flow balance (b), as well as biomass synthesis (a,b) in full and partial denitrification; Table S1: The sequences of primers used in this study; Table S2: Richness and diversity of the biofilms taken from each stage in the MBfR revealed by Illumina high-throughput sequencing analysis Table S3: The abundance of known denitrifiers in each sample (% of total reads). References [11,15,23,24,44,45,46,47] are cited in the supplementary materials.

## References

[1] Du R., Cao S.B., Peng Y.Z., Zhang H.Y., Wang S.Y. (2019). Combined Partial Denitrification (PD)-Anammox: A method for high nitrate wastewater treatment. Environ. Int..

[2] Lycus P., Bothun K.L., Bergaust L., Shapleigh J.P., Bakken L.R., Frostegard A. (2017). Phenotypic and genotypic richness of denitrifiers revealed by a novel isolation strategy. ISME J..

[3] Cao S.B., Du R., Li B.K., Wang S.Y., Ren N.Q., Peng Y.Z. (2017). Nitrite production from partial-denitrification process fed with low carbon/nitrogen (C/N) domestic wastewater: Performance, kinetics and microbial community. Chem. Eng. J..

[4] Li W., Li H., Liu Y.D., Zheng P., Shapleigh J.P. (2018). Salinity-Aided Selection of Progressive Onset Denitrifiers as a Means of Providing Nitrite for Anammox. Environ. Sci. Technol..

[5] Yu C., Qiao S., Yang Y., Jin R.F., Zhou J.T., Rittmann B.E. (2019). Energy recovery in the form of N_2_O by denitrifying bacteria. Chem. Eng. J..

[6] Tang Y.N., Zhou C., Ziv-El M., Rittmann B.E. (2011). A pH-control model for heterotrophic and hydrogen-based autotrophic denitrification. Water Res..

[7] Lee K.C., Rittmann B.E. (2003). Effects of pH and precipitation on autohydrogenotrophic denitrification using the hollow-fiber membrane-biofilm reactor. Water Res..

[8] Tang Y.N., Zhou C., Van Ginkel S.W., Ontiveros-Valencia A., Shin J., Rittmann B.E. (2012). Hydrogen permeability of the hollow fibers used in H-2-based membrane biofilm reactors. J. Membr. Sci..

[9] Ziv-El M.C., Rittmann B.E. (2009). Systematic evaluation of nitrate and perchlorate bioreduction kinetics in groundwater using a hydrogen-based membrane biofilm reactor. Water Res..

[10] Moreno-Vivian C., Cabello P., Martinez-Luque M., Blasco R., Castillo F. (1999). Prokaryotic nitrate reduction: Molecular properties and functional distinction among bacterial nitrate reductases. J. Bacteriol..

[11] Flanagan D.A., Gregory L.G., Carter J.P., Karakas-Sen A., Richardson D.J., Spiro S. (1999). Detection of genes for periplasmic nitrate reductase in nitrate respiring bacteria and in community DNA. FEMS Microbiol. Lett..

[12] Levy-Booth D.J., Prescott C.E., Grayston S.J. (2014). Microbial functional genes involved in nitrogen fixation, nitrification and denitrification in forest ecosystems. Soil Biol. Biochem..

[13] Zhang K.Y., Gu J., Wang X.J., Zhang X., Hu T., Zhao W.Y. (2019). Analysis for microbial denitrification and antibiotic resistance during anaerobic digestion of cattle manure containing antibiotic. Bioresour. Technol..

[14] Chee-Sanford J.C., Connor L., Krichels A., Yang W.H., Sanford R.A. (2020). Hierarchical detection of diverse Clade II (atypical) *nosZ* genes using new primer sets for classical- and multiplex PCR array applications. J. Microbiol. Meth..

[15] Chon K., Chang J.S., Lee E., Lee J., Ryu J., Cho J. (2011). Abundance of denitrifying genes coding for nitrate (*narG*), nitrite (*nirS*), and nitrous oxide (*nosZ*) reductases in estuarine versus wastewater effluent-fed constructed wetlands. Ecol. Eng..

[16] Ge S.J., Peng Y.Z., Wang S.Y., Lu C.C., Cao X., Zhu Y.P. (2012). Nitrite accumulation under constant temperature in anoxic denitrification process: The effects of carbon sources and COD/NO3-N. Bioresour. Technol..

[17] Du R., Cao S.B., Li B.K., Niu M., Wang S.Y., Peng Y.Z. (2017). Performance and microbial community analysis of a novel DEAMOX based on partial-denitrification and anammox treating ammonia and nitrate wastewaters. Water Res..

[18] Le T., Peng B., Su C.Y., Massoudieh A., Torrents A., Al-Omari A., Murthy S., Wett B., Chandran K., DeBarbadillo C. (2019). Impact of carbon source and COD/N on the concurrent operation of partial denitrification and anammox. Water Environ. Res..

[19] Si Z., Peng Y.Z., Yang A.M., Zhang S.J., Li B.K., Wang B., Wang S.Y. (2018). Rapid nitrite production via partial denitrification: Pilot-scale operation and microbial community analysis. Environ. Sci.-Water Res..

[20] Hussain S., Min Z., Zhu X.X., Khan M.H., Li L.F., Cao H. (2019). Significance of Fe(II) and environmental factors on carbon-fixing bacterial community in two paddy soils. Ecotoxicol. Environ. Saf..

[21] Benson A.A., Nonomura A.M., Gerard V.A. (2009). The Path of Carbon in Photosynthesis. XXV. Plant and Algal Growth Responses to Glycopyranosides. J. Plant Nutr..

[22] Tabita F.R. (1999). Microbial ribulose 1,5-bisphosphate carboxylase/oxygenase: A different perspective. Photosynth. Res..

[23] Campbell B.J., Cary S.C. (2004). Abundance of reverse tricarboxylic acid cycle genes in free-living microorganisms at deep-sea hydrothermal vents. Appl. Environ. Microb..

[24] Nanba K., King G.M., Dunfield K. (2004). Analysis of facultative lithotroph distribution and diversity on volcanic deposits by use of the large subunit of ribulose 1,5-bisphosphate carboxylase/oxygenase. Appl. Environ. Microb..

[25] Huang X.Z., Wang C., Liu Q., Zhu Z.K., Lynn T.M., Shen J.L., Whiteley A.S., Kumaresan D., Ge T.D., Wu J.S. (2018). Abundance of microbial CO_2_-fixing genes during the late rice season in a long-term management paddy field amended with straw and straw-derived biochar. Can. J. Soil Sci..

[26] Xia S.Q., Wang C.H., Xu X.Y., Tang Y.N., Wang Z.W., Gu Z.L., Zhou Y. (2015). Bioreduction of nitrate in a hydrogen-based membrane biofilm reactor using CO_2_ for pH control and as carbon source. Chem. Eng. J..

[27] Chung J., Nerenberg R., Rittmann B.E. (2006). Bio-reduction of soluble chromate using a hydrogen-based membrane biofilm reactor. Water Res..

[28] APHA (2005). Standard Methods for Water and Wastewater Examination.

[29] Xia S.Q., Zhang Y.H., Zhong F.H. (2009). A continuous stirred hydrogen-based polyvinyl chloride membrane biofilm reactor for the treatment of nitrate contaminated drinking water. Bioresour. Technol..

[30] Rittmann B.E., McCarty P.L. (2020). Environmental Biotechnology: Principles and Applications.

[31] Xia S.Q., Wu C.Y., Yang X.X., Zhou Y., Zhou L.M., Ran Y.J., Rittmann B.E. (2020). Bioreduction of nitrate in high-sulfate water using a hydrogen-based membrane biofilm reactor equipped with a separate carbon dioxide module. Chem. Eng. J..

[32] McNamara D.P., Whitney K.M., Goss S.L. (2003). Use of a physiologic bicarbonate buffer system for dissolution characterization of ionizable drugs. Pharm. Res..

[33] Qian W.T., Ma B., Li X.Y., Zhang Q., Peng Y.Z. (2019). Long-term effect of pH on denitrification: High pH benefits achieving partial-denitrification. Bioresour. Technol..

[34] Zhang Z.Z., Zhang Y., Chen Y.G. (2020). Comparative Metagenomic and Metatranscriptomic Analyses Reveal the Functional Species and Metabolic Characteristics of an Enriched Denitratation Community. Environ. Sci. Technol..

[35] Hwang J.H., Cicek N., Oleszkiewicz J.A. (2009). Inorganic precipitation during autotrophic denitrification under various operating conditions. Environ. Technol..

[36] Van Ginkel S.W., Kim B.O., Yang Z.M., Sittmann R., Sholin M., Micelli J., Rittmann B.E. (2012). Effect of NaCl on nitrate removal from ion-exchange spent brine in the membrane biofilm reactor (MBfR). Water Sci. Technol..

[37] Cao S.B., Wang S.Y., Peng Y.Z., Wu C.C., Du R., Gong L.X., Ma B. (2013). Achieving partial denitrification with sludge fermentation liquid as carbon source: The effect of seeding sludge. Bioresour. Technol..

[38] Du R., Peng Y.Z., Cao S.B., Li B.K., Wang S.Y., Niu M. (2016). Mechanisms and microbial structure of partial denitrification with high nitrite accumulation. Appl. Microbiol. Biotechnol..

[39] Zumft W.G. (1997). Cell biology and molecular basis of denitrification. Microbiol. Mol. Biol. Rev..

[40] Li W., Shan X.Y., Wang Z.Y., Lin X.Y., Li C.X., Cai C.Y., Abbas G., Zhang M., Shen L.D., Hu Z.Q. (2016). Effect of self-alkalization on nitrite accumulation in a high-rate denitrification system: Performance, microflora and enzymatic activities. Water Res..

[41] Joshi H.M., Tabita F.R. (1996). A global two component signal transduction system that integrates the control of photosynthesis, carbon dioxide assimilation, and nitrogen fixation. Proc. Natl. Acad. Sci. USA.

[42] Baker S.H., Jin S.M., Aldrich H.C., Howard G.T., Shively J.M. (1998). Insertion mutation of the form I cbbL gene encoding ribulose bisphosphate carboxylase/oxygenase (RuBisCO) in Thiobacillus neapolitanus results in expression of form II RuBisCO, loss of carboxysomes, and an increased CO_2_ requirement for growth. J. Bacteriol..

[43] Liu B.B., Mao Y.J., Bergaust L., Bakken L.R., Frostegard A. (2013). Strains in the genus Thauera exhibit remarkably different denitrification regulatory phenotypes. Environ. Microbiol..

[44] Throback I.N., Enwall K., Jarvis A., Hallin S. (2004). Reassessing PCR primers targeting *nirS*, *nirK* and *nosZ* genes for community surveys of denitrifying bacteria with DGGE. FEMS Microbiol. Ecol..

[45] Braker G., Fesefeldt A., Witzel K.P. (1998). Development of PCR primer systems for amplification of nitrite reductase genes (*nirK* and *nirS*) to detect denitrifying bacteria in environmental samples. Appl. Environ. Microb..

[46] Braker G., Tiedje J.M. (2003). Nitric oxide reductase (*norB*) genes from pure cultures and environmental samples. Appl. Environ. Microb..

[47] Lopez-Gutierrez J.C., Henry S., Hallet S., Martin-Laurent F., Catroux G., Philippot L. (2004). Quantification of a novel group of nitrate-reducing bacteria in the environment by real-time PCR. J. Microbiol. Meth..

