# Supplementary material for: Synergistic Inorganic Carbon and Denitrification Genes Contributed to Nitrite Accumulation in a Hydrogen-Based Membrane Biofilm Reactor
Source: Bioengineering (Basel). 2022 May 20;9(5):222. doi: 10.3390/bioengineering9050222 (PMC9137978; doi:10.3390/bioengineering9050222)
Supplement: Supplementary file 1 [file bioengineering-09-00222-s001.zip › bioengineering-1713742-supplementary.pdf]

bioengineering

Supporting information

Synergistic Inorganic Carbon and Denitrification Genes Contributed to Nitrite Accumulation in a Hydrogen-Based Membrane Biofilm Reactor

Si Pang [1,2], Bruce E. Rittmann [3], Chengyang Wu [1,2], Lin Yang [1,2], Jingzhou Zhou [1,2] and Siqing Xia [1,2,*]

1 State Key Laboratory of Pollution Control and Resource Reuse, College of Environmental Science and Engineering, Tongji University, Shanghai 200092, China; pancy@tongji.edu.cn (S.P.); chengyang@tongji.edu.cn (C.W.); jade_ylin@tongji.edu.cn (L.Y.); 1910545@tongji.edu.cn (J.Z.)
2 Shanghai Institute of Pollution Control and Ecological Security, Shanghai 200092, China
3 Biodesign Swette Center for Environmental Biotechnology, Arizona State University, Tempe, AZ 85287, USA; rittmann@asu.edu
* Correspondence: siqingxia@tongji.edu.cn; Tel.: +86-21-65980440

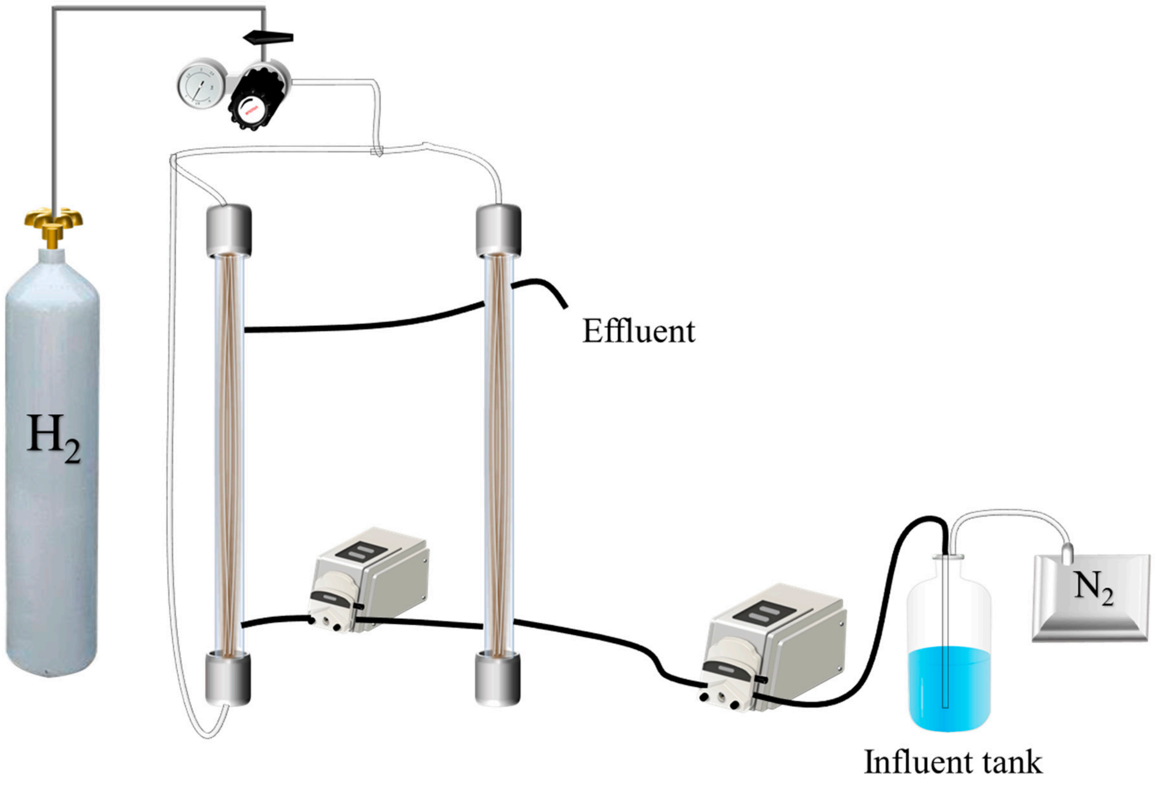

Figure S1. Schematic of the H_2-based MBfR using polypropylene hollow-fiber membranes.

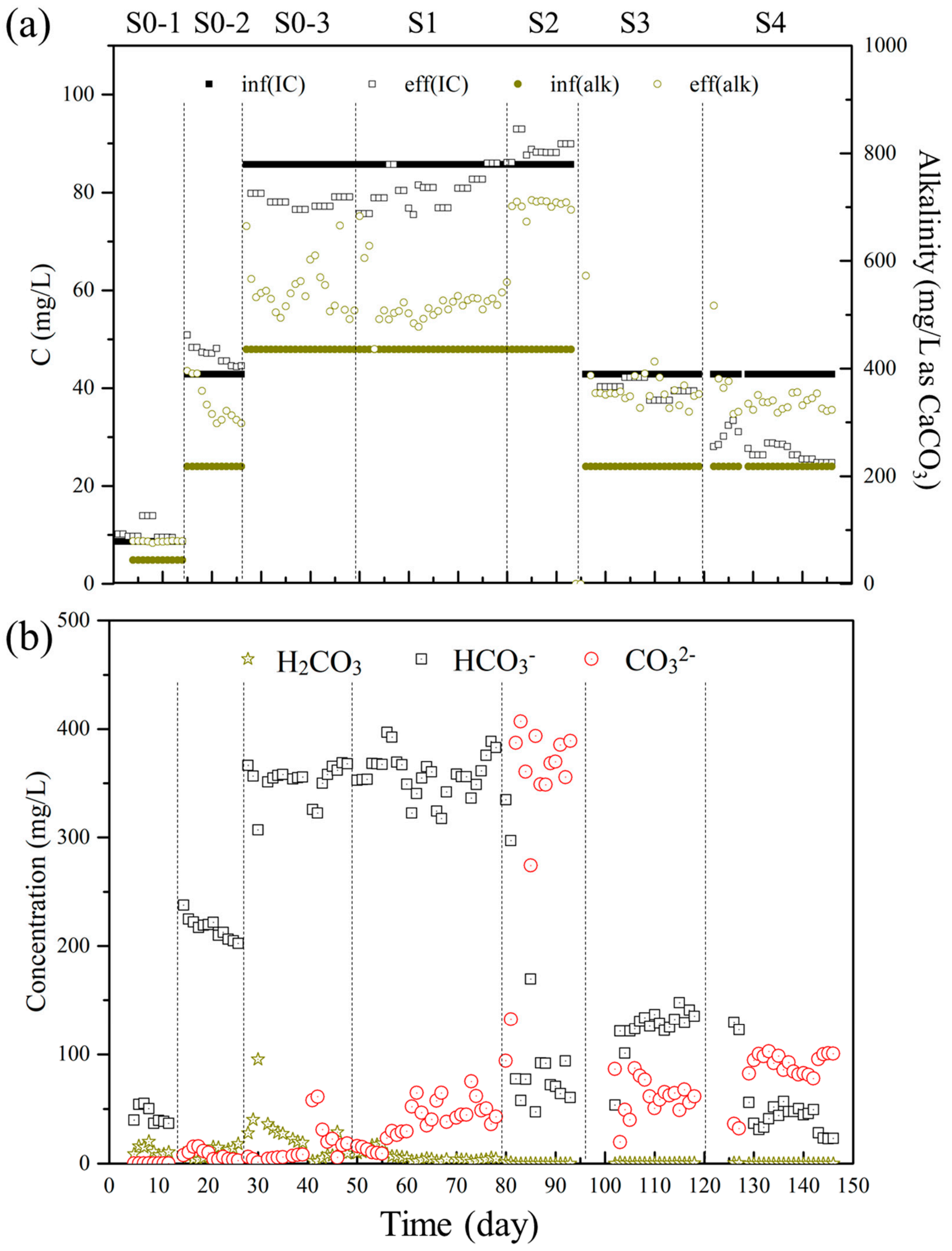

Figure S2. (a) Concentration of influent IC and alkalinity, along with effluent IC and alkalinity, at each stage. (b) Evaluated concentrations of H_2CO_3, HCO_3^-, and CO_3^{2-} at each stage.

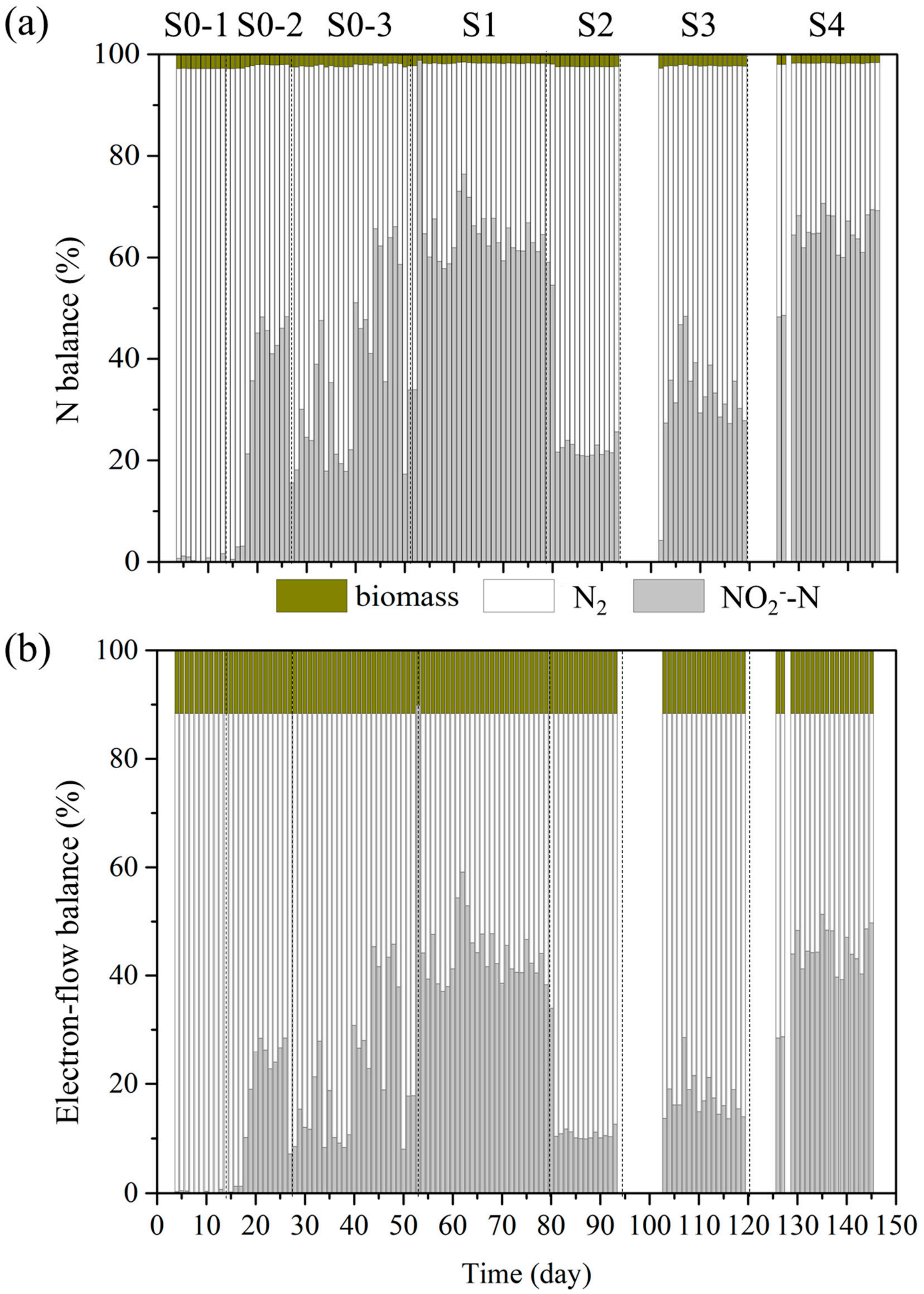

Figure S3. The percentages of N-flow balance (a) and electron-flow balance (b), as well as biomass synthesis (a,b), in full and partial denitrification.

Table S1. The sequences of primers used in this study.

Genes	Primer	Sequence (5'-3')	Concentration used in this study (μmol/L)	Reference
nirS	NirS cd3F	GTSAACGTSAAGGARACSGG	10	1
	NirS 3cdR	GASTTCGGRTGSGTCTTGA		
nirK	nirK 1F	GGMATGGTKCCSTGGCA	10	2
	nirK 5R	GCCTCGATCAGRTTRTGGTT		
cnorB	cnorB-2F	GACAAGNNNTACTGGTGGT	10	3
	cnorB-6R	GAANCCCCANACNCCNGC		
nosZ	nosZf	AGAACGACCAGCTGATCGACA	10	4
	nosZr	TCCATGGTGACGCCGTGGTTG		
napA	NapAV16	GCNCCNTGYMGNTTYTGYGG	10	5
	NapAV66	DATNGGRTGCATYTCNGCCATRT		
narG	1960m2f	TAYGTSGGGCAGGARAAACTG	10	6
	2050m2r	CGTAGAAGAAGCTGGTGCTGTT		
cbbL	K2f	ACCAYCAAGCCSAAGCTSGG	10	7
	V2r	GCCTTCSAGCTTGCCSACCRC		
cbbM	cbbM F	TTCTGGCTGGGBGGHGAYTTYATYAARAAYGACGA	10	8
	cbbM R	CCGTGRCCRGCVCGRTGGTARTG		

Table S2. Richness and diversity of the biofilms taken from each stage in the MBfR, revealed by Illumina high-throughput sequencing analysis.

sample	DNA concentration (ng/μL)	Sequences	0.97 similarity					
			OTUs	Shannon	Simpson	ACE	Chao	Coverage
**S0-1**	**14.1**	38485	351	4.26	0.034	358	356	0.999
S1	80.9	31502	209	2.95	0.114	248	245	0.998
S2	10.7	35308	158	1.74	0.288	201	203	0.998
S3	74.7	41512	250	2.16	0.229	286	279	0.999
S4	125.8	34383	204	2.18	0.207	267	2566	0.998

Table S3. The abundance of known denitrifiers in each sample (% of total reads).

Genus	S0-1	S1	S2	S3	S4
Azoarcus	0.00	0.45	1.54	41.30	31.26
Thauera	0.00	18.02	40.59	8.60	30.10
Hydrogenophaga	0.79	0.88	1.67	3.51	7.09
Alishewanella	0.00	0.11	32.75	18.91	5.71
Unclassified_ Cyclobacteriaceae	0.00	0.00	0.00	3.31	1.02
Bacillus	0.03	3.98	0.01	0.00	0.00
Xanthobacter	1.04	8.02	0.06	0.05	0.04
Dechloromonas	6.09	0.39	0.05	0.00	0.00
No-rank_ Xanthobacteraceae	1.14	0.20	0.12	0.09	0.13
Rhodococcus	1.14	0.00	0.00	0.00	0.00

References

1. Throback, I.N.; Enwall, K.; Jarvis, A.; Hallin, S. Reassessing PCR primers targeting nirS, nirK and nosZ genes for community surveys of denitrifying bacteria with DGGE. *FEMS Microbiol. Ecol.* **2004**, *49*, 401–417.
2. Braker, G.; Fesefeldt, A.; Witzel, K.P. Development of PCR primer systems for amplification of nitrite reductase genes (nirK and nirS) to detect denitrifying bacteria in environmental samples. *Appl. Environ. Microb.* **1998**, *64*, 3769–3775.
3. Braker, G.; Tiedje, J.M. Nitric oxide reductase (norB) genes from pure cultures and environmental samples. *Appl. Environ. Microb.* **2003**, *69*, 3476–3483.
4. Chon, K.; Chang, J.S.; Lee, E.; Lee, J.; Ryu, J.; Cho, J. Abundance of denitrifying genes coding for nitrate (narG), nitrite (nirS), and nitrous oxide (nosZ) reductases in estuarine versus wastewater effluent-fed constructed wetlands. *Ecol. Eng.* **2011**, *37*, 64–69.
5. Flanagan, D.A.; Gregory, L.G.; Carter, J.P.; Karakas-Sen, A.; Richardson, D.J.; Spiro, S. Detection of genes for periplasmic nitrate reductase in nitrate respiring bacteria and in community DNA. *FEMS Microbiol. Lett.* **1999**, *177*, 263–270.
6. Lopez-Gutierrez, J.C.; Henry, S.; Hallet, S.; Martin-Laurent, F.; Catroux, G.; Philippot, L. Quantification of a novel group of nitrate-reducing bacteria in the environment by real-time PCR. *J. Microbiol. Meth.* **2004**, *57*, 399–407.
7. Nanba, K.; King, G.M.; Dunfield, K. Analysis of facultative lithotroph distribution and diversity on volcanic deposits by use of the large subunit of ribulose 1,5-bisphosphate carboxylase/oxygenase. *Appl. Environ. Microb.* **2004**, *70*, 2245–2253.
8. Campbell, B.J.; Cary, S.C. Abundance of reverse tricarboxylic acid cycle genes in free-living microorganisms at deep-sea hydrothermal vents. *Appl. Environ. Microb.* **2004**, *70*, 6282–6289.
